# DNA metabarcoding reveals a broad dietary range for Tasmanian devils introduced to a naive ecosystem

**DOI:** 10.1002/ece3.8936

**Published:** 2022-05-19

**Authors:** Elspeth A. McLennan, Phil Wise, Andrew V. Lee, Catherine E. Grueber, Katherine Belov, Carolyn J. Hogg

**Affiliations:** ^1^ School of Life and Environmental Sciences University of Sydney Sydney New South Wales Australia; ^2^ Save the Tasmanian Devil Program NRE Hobart Tasmania Australia

**Keywords:** assisted colonisation, carnivore, Diet, metabarcoding

## Abstract

Top carnivores are essential for maintaining ecosystem stability and biodiversity. Yet, carnivores are declining globally and current *in situ* threat mitigations cannot halt population declines. As such, translocations of carnivores to historic sites or those outside the species’ native range are becoming increasingly common. As carnivores are likely to impact herbivore and small predator populations, understanding how carnivores interact within an ecosystem following translocation is necessary to inform potential remedial management and future translocations. Dietary analyses provide a preliminary assessment of the direct influence of translocated carnivores on a recipient ecosystem. We used a metabarcoding approach to quantify the diet of Tasmanian devils introduced to Maria Island, Tasmania, a site outside the species’ native range. We extracted DNA from 96 scats and used a universal primer set targeting the vertebrate 12S rRNA gene to identify diet items. Tasmanian devils on Maria Island had an eclectic diet, with 63 consumed taxa identified. Cat DNA was detected in 14% of scats, providing the first instance of cats appearing as part of Tasmanian devil diets either via predation or scavenging. Short‐tail shearwaters and little penguins were commonly consumed, corresponding with previous surveys showing sharp population declines in these species since the introduction of Tasmanian devils. Our results indicate that the introduction of carnivores to novel ecosystems can be very successful for the focal species, but that commonly consumed species should be closely monitored to identify any vulnerable species in need of remedial management.

## Graphical abstract

Top‐order carnivores are essential for maintaining ecosystem stability and biodiversity. We used a metabarcoding and next‐generation sequencing approach to quantify the diet of Tasmanian devils introduced to Maria Island, Tasmania, a site outside the species’ native range. Our results indicate that the translocation of carnivores to vacant ecosystems can be very successful, but that dietary preferences should be closely monitored to identify any vulnerable species in need of remedial management.

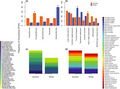

## 1 INTRODUCTION

The global decline of top carnivores is contributing to the biodiversity crisis (Ritchie & Johnson, 2009). Top carnivores are crucial for maintaining ecosystem stability via top‐down control, through predation and suppression of herbivores and competition with meso‐predators (Estes et al., 2011). Disruptions to such interactions can cause overgrazing by herbivores and over‐predation of small prey by meso‐predators (Wallach et al., 2010). Perturbations to ecosystem stability have highlighted the critical role carnivores play in maintaining healthy environments (Ritchie & Johnson, 2009), prompting large‐scale conservation programs for these species and their subsequent ecological influence (Wolf & Ripple, 2018).

The Endangered Tasmanian devil (*Sarcophilus harrisii*; IUCN, 2022), hereafter referred to as “devil”, is a top carnivore of the Tasmanian ecosystem. In the last 20 years, devil populations have experienced an 80% decline due to the emergence of an infectious cancer, devil facial tumor disease (Hawkins et al., 2006; Lazenby et al., 2018; McCallum et al., 2009). The ecosystem consequences of devil declines across Tasmania are currently under investigation. Notably, feral cat (*Felis catus*) sightings have increased at sites where devils have declined (Cunningham et al., 2019). Cats severely impact native Australian species, particularly small herbivores and birds, by consuming an estimated 459 million individuals per year across Australia (Murphy et al., 2019). To preserve devils, and their ecological role of meso‐predator suppression, the Tasmanian and Australian Federal governments launched the Save the Tasmanian Devil Program (STDP) in 2003. As part of the formal conservation response, devils were introduced to Maria Island National Park, Tasmania during an assisted colonization to create a free‐range, disease‐free population in 2012 (Wise et al., 2019). Ecological risk assessments, considering the species most vulnerable to a devil translocation, suggested the devil carrying capacity of the island to be 100–120 individuals (Jones & McCallum, 2007; STDP pers comm). This number reflects a point where devils may start to have a negative impact on resident species of the island, rather than where devils would succumb to density‐dependent pressures (STDP pers comm). As of November 2017, the date of final sample collection for this study, the population size of devils on Maria Island was estimated at 103 (95% CI 87–133; STDP unpublished data).

Devils are generalists and scavengers, commonly consuming marsupials, rodents, livestock species, birds, and fish (Pemberton et al., 2008). The STDP identified several species at risk from devil predation on Maria Island, namely little penguins (*Eudyptula minor*) and short‐tailed shearwaters (*Puffinus tenuirostris*; STDP, 2012). These species are not of conservation concern globally (IUCN, 2022) and have large, healthy colonies on neighboring islands. Impacts from devil introductions were anticipated and the assisted colonization was approved given the conservation benefits of translocation to the Endangered devil (Wise et al., 2019). While devil conservation was the primary goal of the assisted colonization to Maria Island, it is necessary to evaluate how a carnivore introduction may impact resident species at the recipient site to inform potential remedial management and future translocations.

One way to measure the direct influence of carnivores is through dietary analysis (Monterroso et al., 2019). Previous dietary assessments of translocated carnivores have used indigestible material, such as bone and feathers, found in scats and stomach contents to assign prey items (e.g., Rapson, 2004; Sankar et al., 2010; West et al., 2019). However, taxonomically assigning hard parts to species level is often not possible (Pompanon et al., 2012), limiting assessment of the full scope of a carnivore's dietary niche (De Barba et al., 2014). Metabarcoding utilizes highly conserved genetic regions with sufficient interspecific variation (such as mitochondrial DNA), termed “barcodes,” to differentiate among species present in a mixed sample (such as a carnivore's scat; Hebert & Gregory, 2005). It allows for detection of species which traditionally do not have ‘hard indigestible parts’ such as invertebrates, reptiles and amphibians (Granquist et al., 2018; Norgaard et al., 2021). However, when assessing carnivore and omnivore diets, the barcodes used to detect vertebrate diet items will also amplify host DNA (Shehzad et al., 2012). This may result in sequences of primarily host DNA, with the importance of common diet items underestimated, and rarer items potentially missed altogether (Green & Minz, 2005). This problem can be overcome by using a blocking oligonucleotide, designed specifically to prevent amplification of the host DNA (Vestheim & Jarman, 2008). Blocking oligonucleotide have successfully inhibited host DNA amplification and improved the amplification of nonhost taxa in several species including the Eurasian otter (*Lutra lutra*; Pertoldi et al., 2021) and wild pig (*Sus scrofau*; Robeson et al., 2018).

While molecular methods have greatly enhanced our ability to detect consumed species, there remains some aspects of an animal's dietary niche that cannot be explored with metabarcoding alone. For instance, it is still only possible to reliably say whether a specimen has been consumed (i.e., presence or absence), not the proportion of a specimen in a scat sample. Attempts to correlate the number of sequence reads with the biomass of a specimen have had variable results, given the risks of amplification bias with unequal primer binding efficiency across species (Alberdi et al., 2017; Elbrecht & Leese, 2015). It is still not possible to ascertain the age, sex, or size of the prey consumed, which would provide very useful information for the potential demographic impacts and management actions for prey species. Nor can current dietary analyses distinguish between predation, scavenging, or meta‐prey, the prey of animals that were consumed by the study species. That being said, metabarcoding can provide measures of commonly consumed taxa across a population (Thuo et al., 2020), demographic and seasonal consumption differences within species (Tang et al., 2021), and when combined with ecological survey data whether any correlated changes to prey density numbers are occurring with commonly consumed taxa (Egeter et al., 2019).

Here, we aimed to provide the first dietary assessment of a translocated carnivore via metabarcoding, to identify commonly consumed taxa which may require remedial management, and to provide insights into the ecological impact of carnivore assisted colonizations, beyond the focal species.

## 2 MATERIALS AND METHODS

### 2.1 Study population and sample collection

**FIGURE 1:**
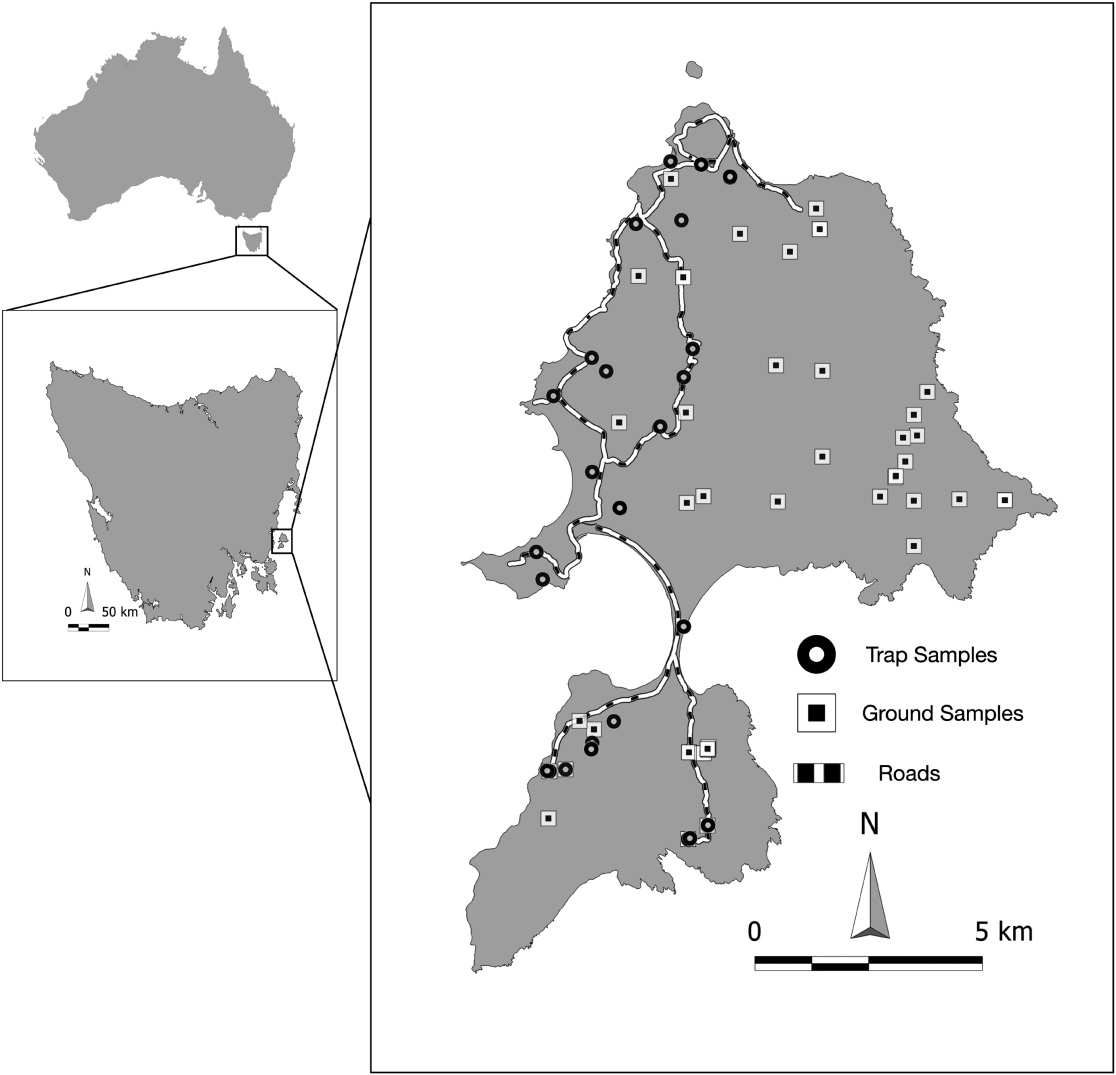
Map of Maria Island, Tasmania showing its relative position within Tasmania and Australia. The circles represent known Tasmanian devil scat samples collected from traps (*N* = 42) and the squares represent Tasmanian devil scat samples collected from the ground (*N* = 54) for the present study (November 2016–November 2017)

All trapping and sampling were undertaken by STDP staff (Department of Natural Resources and Environment) and volunteers in accordance with the STDP’s *Standard Operating Procedure for Trapping and Handling Wild Devils* (see Figure 1 for sample locations). Between November 2016 and November 2017, a total of 96 scat samples were collected both directly from devil traps and from the ground. For samples collected directly from devils (*N* = 42), baited‐trapping using PVC pipe traps, as described in Hawkins et al. (2006), was conducted over one six‐night (January 2017) and one 12‐night (May 2017) trapping trip.

Traps were located primarily on the western side of the island (for trap locations see figure 1 in McLennan et al. (2018)). Individuals were identified using subcutaneous microchips (ISO transponder, Allflex Pty Ltd, Australia). Scats were collected either from the PVC pipe trap, or the hessian bag used when processing devils. Samples were placed into zip‐lock bags, labeled with the devil's name, date, and trap location and stored at −20°C. Five of the 42 samples collected directly from traps were repeat samples of the same individuals at two different timepoints. Two samples were sampled three times at different time points.

Ground samples (*N* = 54) were collected during the January and May 2017 trapping trips (described above) as well as the quarterly camera trap trips (February, April, August and November 2016 and 2017). Camera traps are distributed across the island so the collection of scats from these sites provided a broader geographical representation of samples than the trapping trips alone. Samples were collected from the ground, placed into zip‐lock bags, and stored at −20°C. Only samples that still contained moisture, as an indication of freshness, were retained for analysis. To confirm that ground samples were from devils, DNA extracts (see below) were genotyped at four microsatellite loci with devil‐specific primers (Sha010, Sha013, Sha014, Sha040; for details on microsatellite typing see McLennan et al. (2018) and references therein). All ground samples were successfully typed at all four microsatellite loci, therefore, designated as devil samples. The microsatellite markers did not have sufficient allelic diversity to assign these samples to known devils on the island. As such, sex, age, and individual ID for these samples were unknown.

For both known and ground samples (Figure 1), there were an approximate even number of samples collected across the warmer (*N* = 50) and cooler (*N* = 46) months. Samples collected in summer (January, February) and spring (November; *N* = 50) are hereafter referred to as “summer” samples and samples collected in winter (May, August) and autumn (April; *N* = 46) are hereafter referred to as “winter” samples.

### 2.2 DNA extraction

All DNA extractions were performed in a fume hood within a laboratory used exclusively for this study. All fecal samples (*N* = 96) were subsampled three times from different places along the scat (0.5 cm from each end and in the center) taking care not to sample the external surface which will have a high concentration of host epithelial cells (Waits & Paetkau, 2005), resulting in 288 extractions from 96 scat samples. This subsampling was to increase the likelihood of all diet items being extracted, as estimates of diet diversity increase with the number of extractions per sample (Mata et al., 2019). DNA was extracted from 180–200 mg of feces using the QIAamp DNA Stool Mini Kit (Qiagen), following the manufacturer's protocol. Extracted DNA was eluted in AE buffer (10 mM Tris‐Cl, 0.5 mM EDTA, pH 0.9; Qiagen) in a final volume of 75 µl. Negative controls were performed with each extraction round to monitor for possible contaminants.

### 2.3 Primer selection and blocking oligonucleotide design and assessment

To identify DNA from vertebrate diet items in devil scats, we used the primer pair 12Sv5F/12Sv5R (Table 1; Riaz et al., 2011) to amplify a ~100‐bp fragment of the V5 loop of the 12S mitochondrial gene. This primer pair was chosen for the present study for its proven use in the detection of vertebrates in other diet studies (De Barba et al., 2014; Kocher et al., 2017; Shehzad et al., 2012).

**TABLE 1:** Primer sequences for 12Sv5 and 12Sv5DevilB. Sequences of the primer pairs used in this study, amplification length of 12Sv5 product is ~100 bp

Name	Primer sequence (5′−3′)	References
12Sv5F	TAGAACAGGCTCCTCTAG	Riaz et al. (2011)
12Sv5R	TTAGATACCCCACTATGC	Riaz et al. (2011)
12Sv5DevilB	ACCCCACTATGCTTGGCCGTAAA[C3]	This study

The 12Sv5DevilB (Table 1) blocking oligonucleotide specific to the devil sequence was designed following Vestheim and Jarman (2008). In essence, the blocking oligonucleotide overlaps where the reverse universal primer would anneal to host DNA and extends into the unique host sequence (Vestheim & Jarman, 2008). The addition of a 3′ C3 spacer prevents elongation during the PCR with minimal impact on the annealing properties of the oligonucleotide (Vestheim & Jarman, 2008). To test the efficacy of 12Sv5DevilB for preventing the amplification of devil, or host DNA, six pilot devil scat samples were extracted, amplified with and without the blocking oligonucleotide and sent for next‐generation sequencing (methods outlined below). Samples from species known to be commonly consumed by devils on Tasmania, Forester kangaroo (*Macropus giganteus*) and Bennett's wallaby (*Macropus rufogriseus*; Pemberton et al., 2008), were amplified with the blocking oligonucleotide using the PCR protocol outlined below to test whether they would fail to amplify. Considering the results from the blocking oligonucleotide trials (see Results), all PCRs for the final samples (*N* = 96; Figure 1) included 12Sv5DevilB (see below).

### 2.4 PCR and next‐generation sequencing for dietary analysis

All DNA amplifications were carried out in a final volume of 25 µl with 12 µl 2× MyTaq PCR Master Mix (containing DNA polymerase, buffer, MgCl_2_, and dNTPs; Bioline, UK), 2.5 µl each of 12Sv5F/R (final concentration 10 µM), 2.5 µl of 12Sv5DevilB (final concentration 100 µM) and 3 µl of template DNA or negative extraction control. Products were amplified using a T100 Thermal Cycler (BioRad) with 10 min for enzyme activation at 95°C, 35 cycles of denaturation at 95°C for 30 s, 30 s of annealing at 50°C, and 30 s of extension at 72°C, followed by a final extension for 10 min at 72°C. Each extraction (*N* = 288; i.e., triplicates of 96 samples) was amplified three times (*N* = 864 amplifications) and then pooled prior to sequencing (*N* = 288). Amplification was confirmed using 1% agarose/TBE gel electrophoresis stained with SYBR safe (Life Technologies), alongside a 1‐kb size standard (Bioline) and run for 45 min at 100 V. Bands were visualized under ultraviolet light using a ChemiDoc XRS+system (BioRad) and images were analyzed with ImageLab (BioRad). DNA concentration was quantified using a Nanodrop 2000 Spectrophotometer (ThermoFisher Scientific).

Amplicon products were sent to The Ramaciotti Centre for Genomics (University of New South Wales, Australia) for library preparation and sequencing. PCR products were indexed, normalized to 1 ng of DNA, and pooled for sequencing using the Nextera XT Index Kit v2 Set C (Illumina) following the manufacturer's instructions. Sequencing was performed using the Illumina NextSeq 500 Sequencing System (Illumina) using a 2 × 100 bp Mid Output sequencing run (Illumina) generating paired‐end reads.

### 2.5 Diet species assignment

Sequence reads were analyzed using the metabarcoding program OBITools (Boyer et al., 2016). First, paired‐end reads were assembled using the “illuminapairedend” function, unassembled sequences were removed using “obigrep” and reads were assigned to samples using “ngsfilter”. The combination of indexes on the forward and reverse primers were used to assign sequences to samples. To account for potential tag‐jumping, “ngsfilter” only allows for a complete match of index combinations to assign sequences (Boyer et al., 2016). Next, the “obiuniq” function was used to dereplicate reads into unique sequences by comparing all reads in the dataset, grouping strictly identical reads together and outputting the sequence for each group and its count in the original dataset, only reads with a count of ≥10 were retained. Then, “obiclean” was used to remove any possible PCR errors (any sequence variants with a count greater than 5% of their own count). A reference database was built by downloading the relevant portion of the vertebrate 12S mitochondrial gene targeted by the 12Sv5 primer pair for all species present in the EMBL nucleotide library using the ecoPCR program (Bellemain et al., 2010; Ficetola et al., 2010). Sequences were assigned to a taxon ID using the “ecotag” function which compares each sequence to the reference database and assigns a record to that taxon which specifies: (1) the percentage of identity between the reference and query sequence, (2) the taxon ID (taxid) or final assignment of the sequence, and (3) the scientific name of the assigned taxid. Finally, the “obitab” function was used to create a tab‐delimited file from a fasta file that was imported to Microsoft Excel for further analysis. Only sequences with ≥94% identity match to a reference sequence in the database were retained for analysis (Xiong et al., 2017).

**FIGURE A1:**
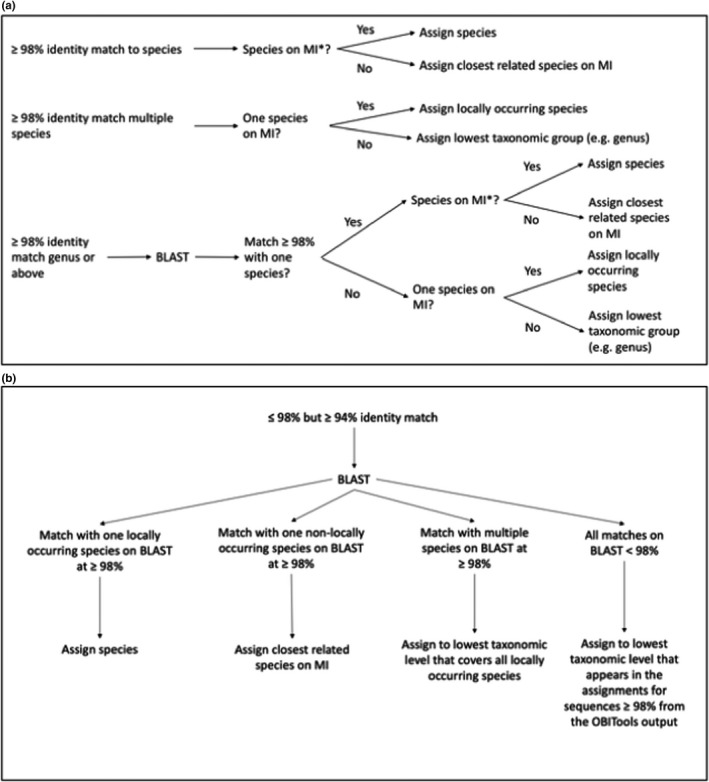
Flowchart outlining how species assignments were made from the raw OBITools output depending on the identity match percentage with the reference database. (a) Decisions were made differently based on if a sequence matched ≥98% to the reference database, or (b) if a sequence matched ≤98% but ≥94% to the reference database. MI = Maria Island

From the OBITools output, extensive survey information carried out by STDP of the locally occurring species on Maria Island was used to validate the taxonomic assignments of the genetic data. This survey combined historical reports and observational surveys from 2010 and 2011. The STDP survey only covered mammals, birds, and reptiles. Any aquatic species that were assigned to sequences were assumed potentially present on Maria Island shores if they occurred in Tasmania. The criteria used to confirm or manually assign sequences to taxa are described in the appendix (Figure A1). Briefly, any sequences that matched to the reference database ≥98% were accepted. Any sequences that matched ≥94% but <98% were run through Basic Local Alignment Search Tool (BLAST; NCBI) and were assigned if they had a ≥ 98% match on BLAST (Figure A1). Only assignments that could be made to the level of order or below were retained. Human and devil sequences were considered contamination and discarded, as there is currently no evidence to suggest that cannibalism exists in devils and current methods would not be able to distinguish between different devil individuals within a single scat.

Devil diets were quantified using frequency of occurrence (FOO) which is the count of scats in which a particular diet item occurred divided by the total number of scats analyzed. Four FOO analyses were undertaken, whereby the number of scats analyzed remained constant (*N* = 96), but the breakdown of taxa used for evaluating the numerator (count of scats in which a diet item occurred) varied according to the taxonomic depth threshold applied, species (*N* = 44), genus (*N* = 48), family (*N* = 41), and order (*N* = 32). The FOO of each taxonomic group were visualized using bar charts in R (R Core Team, 2019). As broad consumption patterns in genera were captured in the species and/or order analysis, and family in the order and/or species analysis (see Results), more in‐depth assessments of devil diets were carried out at the species and order levels. Both species and order FOO analyses were further separated into summer (*N* = 50) and winter samples (*N* = 46). To assess any seasonal differences in species of interest consumed across summer and winter, Pearson's chi‐square tests were applied. Following guidelines for the Pearson's chi‐square test, only cell counts greater than five were included, enabling seasonal comparison of eight species and five orders. To overcome potential problems associated with multiple testing, a sequential Holm–Bonferroni correction was applied with a significance threshold of α < .05 (Abdi, 2010). Stacked bar charts were generated in R to visualize the FOO values of diet items assessed between summer and winter at the order and species level, separated by season.

## 3 RESULTS

### 3.1 Blocking oligonucleotide assessment

**FIGURE 2:**
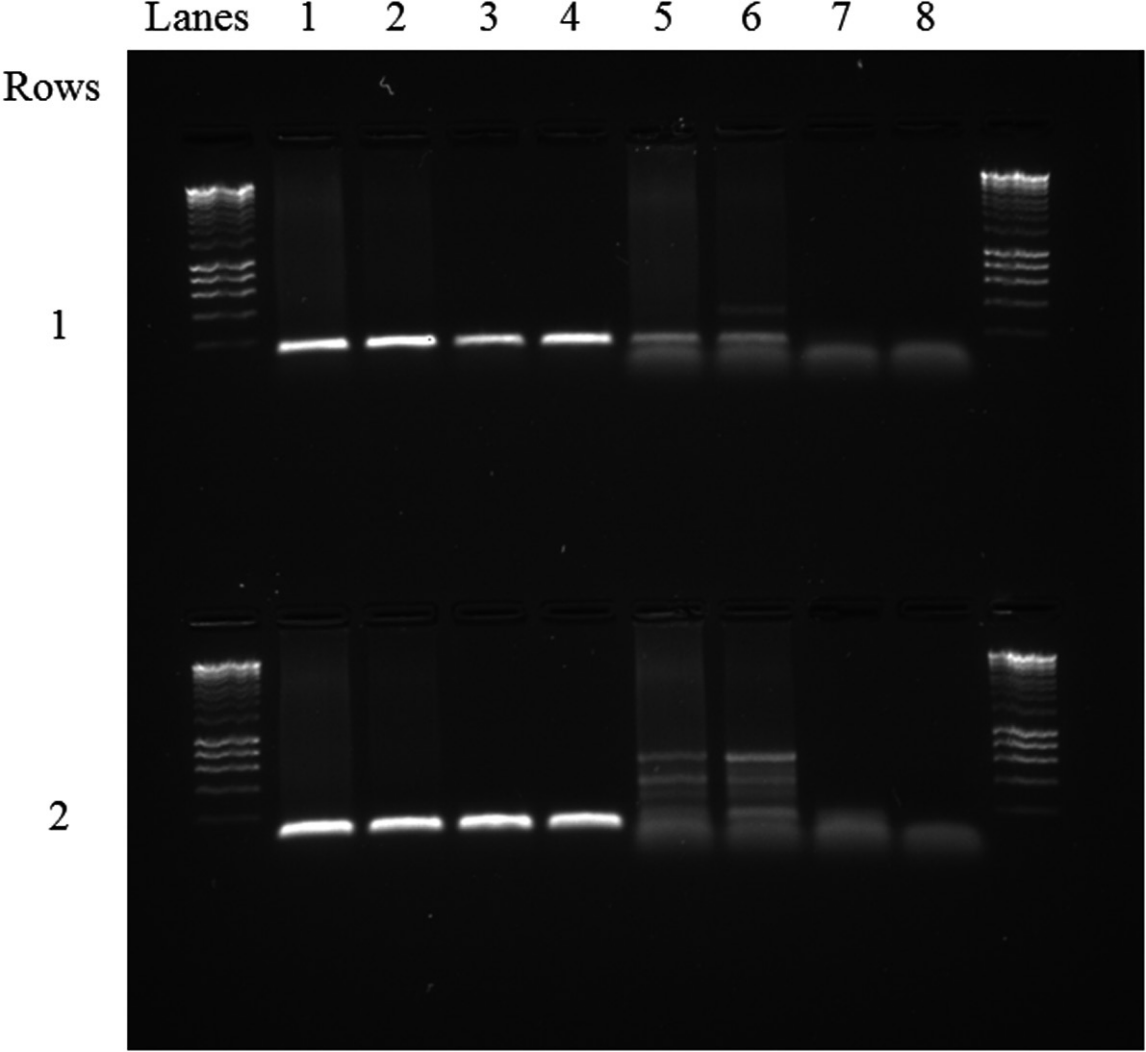
1% TBE agarose gel, stained with SYBR safe, showing amplicon products of Bennett's wallaby (*Macropus rufogriseus*) with (row 1 lanes 1–2) and without (row 1 lanes 3–4) the addition of 12Sv5DevilB (Tasmanian devil blocking oligonucleotide) and Forester kangaroo (*Macropus giganteus*) with (row 2, lanes 1–2) and without (row 2, lanes 3–4) the additional of 12Sv5DevilB. For comparison, lanes 5–8 in both rows contain the amplicon products of Tasmanian devil DNA amplified with the 12Sv5 primer pair and 12Sv5DevilB. This gel shows that 12Sv5DevilB did not block amplification of Bennett's wallaby or Forester kangaroo DNA at 12Sv5 but was successful in reducing Tasmanian devil DNA amplification

To test whether the devil blocking oligonucleotide would prevent amplification of other marsupial species, samples from Forester kangaroo and Bennett's wallaby were amplified with 12Sv5F/R and 12Sv5DevilB (see Methods above). 12Sv5DevilB did not suppress amplification of the two macropod species DNA (Figure 2). To test whether the primers would behave in the same way in a pooled sample, six pilot samples were amplified with and without the blocking oligonucleotide and sequenced (following the Methods reported above). When pilot samples (*N* = 6) were amplified without the 12Sv5DevilB blocking oligonucleotide, devil sequences represented 57% (*N* = 863,528) of the total read count (total count = 1,514,962; average per individual = 252,494 [±126,927.1 SD]). Inclusion of the 12Sv5DevilB blocking oligonucleotide reduced devil sequences to 7% (*N* = 120,642) of the total read count (total count =1,723,468; average per individual = 287,245 [±145,112.5 SD]). As all taxa identified without the blocking oligonucleotide were also identified with its inclusion, as well as one additional taxon, we determined that the inclusion of the blocking oligonucleotide did not limit our ability to detect any taxa. As such, samples for the main study (*N* = 96) were amplified with the blocking oligonucleotide.

### 3.2 Taxa assignment

We generated 16,082,398 sequences across 288 samples (triplicates of 96 scats). After filtering with OBITools, we obtained 10,215 unique sequences; manual sequence assignment and consolidation of triplicate samples reduced these further to 704 unique sequences. Of the final assigned sequences (*N* = 704), 356 (50.6%) had identity matches to the reference database of ≥98% and 348 (49.4%) had identity matches to the reference database of ≤98% but ≥94%.

**FIGURE 3:**
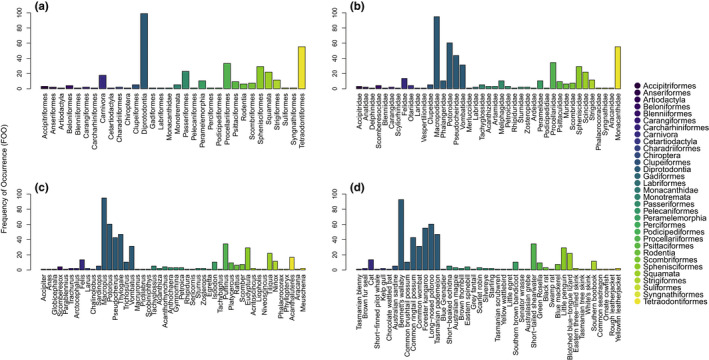
Frequency of occurrence (FOO) for orders (a), families (b), genera (c), and species (d) consumed by Tasmanian devils on Maria Island. Each family, genera, and species are colored by the order they belong to as indicated by the legend

DNA from 63 taxa (i.e., presumed diet items) were identified in the scats of devils on Maria Island. Of these, 44 (70%) were assigned to the species level, nine (14%) were assigned to genus, eight (13%) were assigned to family, and two (3%) were assigned to order (Figure 3). The 44 assigned species included 16 birds, nine fish, eight marsupials, six eutherian mammals, four reptiles, and one monotreme. The nine assigned genera included five bird genera, three fish genera, and one mammal genus. The eight assigned families included five bird families, two fish families and one mammal family. The two assigned orders included one marsupial and one bird order.

### 3.3 Commonly consumed taxa

A maximum of 17 taxa were observed in a single scat, with an average of 7.4 taxonomically distinct diet items (±2.9 SD) per scat (*N* = 95 scats). The average number of diet items identified between ground and trap samples was comparable, 7.6 (±3.3 SD) and 7.3 (±2.4 SD), respectively. We were unable to assign any diet items in one sample, across all three of this sample's extraction replicates, as all sequences were too low quality and filtered out during the OBITools pipeline.

Of the 32 orders consumed by devils, four were commonly consumed (FOO ≥25%; Figure 3a). Considering all assignments together, the order Diprotodontia (an order of marsupials including macropods and others) dominated the diet of devils on Maria Island (diprotodonts; 99% FOO; Figure 3a), followed by Tetraodontiformes (an order of ray‐finned fishes, including leatherjackets [family Monacanthidae]; 55% FOO; Figure 3a), and Procellariiformes (an avian order including shearwaters, petrels and albatrosses; 33% FOO; Figure 3a). The most consumed diet items assigned at the family level (*N* = 41) were Macropodidae (macropods [order: Diprotodontia]; 95% FOO; Figure 3b), Potoroidae (potoroos and bettongs [order: Diprotodontia]; 60% FOO; Figure 3b), Monacanthidae (leatherjackets [order: Tetraodontiformes]; 55% FOO; Figure 3b), Pseudocheiridae (ring‐tail possums [order: Diprotodontia]; 44% FOO; Figure 3b), Procellariidae (shearwaters, petrels and prions [order: Procellariiformes]; 34% FOO; Figure 3b), Vombatidae (wombats [order: Diprotodontia]; 31% FOO; Figure 3b), and Spheniscidae (penguins [order: Sphenisciformes]; 29% FOO; Figure 3b). The most common diet items assigned at the genus level (*N* = 48) were Macropus (kangaroos and wallabies [order: Diprotodontia], FOO 95%; Figure 3c), Potorous (potoroos [order: Diprotodontia], FOO 60%; Figure 3c), Thylogale (pademelons [order: Diprotodontia], FOO 47%; Figure 3c), Pseudocheirus (ring‐tail possums [order: Diprotodontia], FOO 43%; Figure 3c), Puffinus (shearwaters [order: Procellariiformes], FOO 34%; Figure 3c), Vombatus (bare‐nosed wombats [order: Diprotodontia], FOO 31%; Figure 3c), and Eudyptula (little penguins [order: Sphenisciformes], FOO 29%; Figure 3c). The most consumed diet items assigned to species on the island were the Diprotodonts Bennett's wallaby (93% FOO Figure 3d), long‐nosed potoroo (*Potorous tridactylus apicalis*; 60% FOO; Figure 3d), and Forester kangaroo (55% FOO; Figure 3d). Of the 44 taxa assigned to species, eight were commonly consumed (FOO ≥25%; Figure 3d). The two species at risk of predation by devils (little penguin and short‐tailed shearwater; STDP, 2012) had an FOO of 29% and 34%, respectively (Figure 3d). All the commonly consumed families and genera were identified both as commonly consumed orders and species, except for penguins which were identified only as commonly consumed at the species level.

### 3.4 Seasonal diet comparisons

**FIGURE 4:**
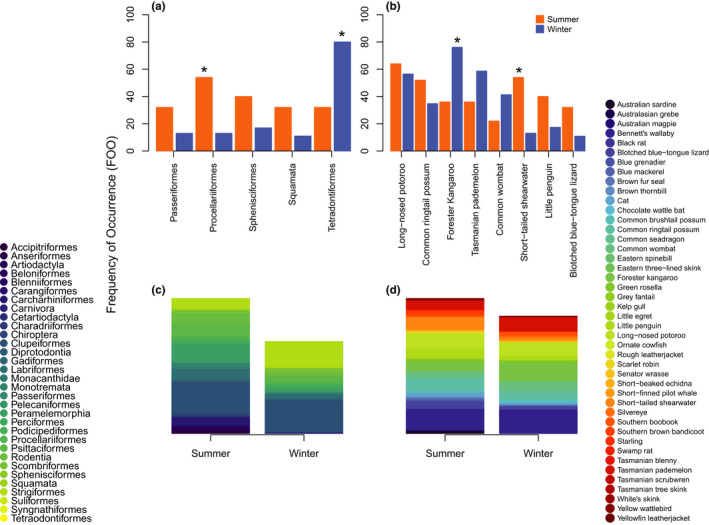
Seasonal differences in diet items (with a frequency of occurrence (FOO) >10 for statistical power) consumed by Tasmanian devils on Maria Island, separated into orders (a) and species (b). A sequential Holm–Bonferroni correction was applied to account for multiple testing. Asterix denotes statistically significant value. Stacked column graphs show the overall consumption differences of orders (c) and species (d) consumed between summer and winter, legend on the left applies to orders (c) and legend on the right applies to species (d)

Summer diets contained 40 species across 28 orders while winter diets contained 26 species across 15 orders; 22 species (from 14 orders) were found in samples from both seasons (Figure 4). Of the orders with sufficient data to perform Pearson's chi‐square tests, Tetraodontiformes were consumed significantly more in winter than summer, and Procellariiformes were consumed significantly more in summer than winter (Table 2). Squamata (scaled reptiles), Sphenisciformes (penguins), and Passeriformes (songbirds) were not differentially consumed across seasons (Table 2). Of the species with sufficient data to perform Pearson's chi‐square tests, short‐tailed shearwaters were consumed more in summer than winter, and Forester kangaroos were consumed more in winter than in summer (Table 2). Blotched blue‐tongue lizards (*Tiliqua nigrolutea*), little penguins, Tasmanian pademelons (*Thylogale billardierii*), common wombats (*Vombatus ursinus*), common ringtail possums (*Pseudocheirus peregrinus*), and long‐nosed potoroos (*Potorous tridactylus*) were not consumed differently across seasons (Table 2).

**TABLE 2:** Test statistics from Pearson's chi‐square analyses for seasonal differences in diet items (with a frequency of occurrence (FOO) >10 for statistical power) consumed by Tasmanian devils on Maria Island. A sequential Holm–Bonferroni correction was applied to account for multiple testing. Taxonomic information for these species can be found in Table A1. Asterix denotes statistically significant value

Diet item	Summer count	FOO	Winter count	FOO	Chi‐square	*p*‐Value
Short‐tailed shearwater *Puffinus tenuirostris*	27	54	6	13	16.046	<.001*
Forester kangaroo *Macropus giganteus*	18	36	35	76	13.99	<.001*
Blotched blue‐tongue lizard *Tiliqua nigrolutea*	16	32	5	11	5.0841	.02415
Little penguin *Eudyptula minor*	20	40	8	17	4.8838	.02711
Tasmanian pademelon *Thylogale billardierii*	18	36	27	59	4.0862	.04324
Common wombat *Vombatus ursinus*	11	22	19	41	3.3057	.06904
Common ringtail possum *Pseudocheirus peregrinus*	26	52	16	35	2.2287	.1355
Long‐nosed potoroo *Potorous tridactylus*	32	64	26	57	0.29119	.5895
Tetraodontiformes	16	32	37	80	20.812	<.001*
Procellariiformes	27	54	6	13	16.046	<.001*
Squamata	16	32	5	11	5.0841	.02415
Sphenisciformes	20	40	8	17	4.8838	.02711
Passeriformes	16	32	6	13	3.8597	.04946

## 4 DISCUSSION

Here, we present the first metabarcoding dietary assessment of a top carnivore introduced beyond its native range. Compared to traditional scat analyses, metabarcoding revealed devils on Maria Island to have an eclectic diet with 63 taxa identified overall (compared to 13–15 taxa identified across 88–183 scats using traditional analyses (Rogers et al., 2016; Wise et al., 2019)), and individuals averaging 7.4 unique diet items. Devils also appear to have a highly varied diet compared to other scavenging and opportunistically predatory carnivores such as coyotes (*Canis latrans*) and *Gyps* vultures, where metabarcoding revealed 18 and 14 diet items consumed, respectively, using the same (98%) or lower (95%) identity match thresholds for assigning species (Ghosh‐Harihar et al., 2019; Shi et al., 2019).

Overall, the most consumed taxa were Diprotodontia (especially macropods), Tetraodontiformes (especially leatherjacket fish), and Procellariiformes (especially short‐tailed shearwaters). Our study was congruent with previous morphological analyses of devil diets, which identified Diprotodontia (59%–88% FOO) and birds (21%–63% FOO) as consistently important diet items for devils across Tasmania and Maria Island (Pemberton et al., 2008; Rogers et al., 2016; Wise et al., 2019). With morphological methods, all feathers and eggshells could only be assigned to class Aves (Pemberton et al., 2008; Rogers et al., 2016; Wise et al., 2019). Here, our metabarcoding and NGS approach enabled us to identify 19 bird species across 11 orders, 11 fish species across 10 orders, and four reptile species from one order, none of which were previously identified with traditional methods (Pemberton et al., 2008; Rogers et al., 2016; Wise et al., 2019). Our results reiterate the power of DNA‐based dietary analyses to provide a deeper understanding of the complexity of generalist carnivore diets. A state‐wide metabarcoding dietary study across Tasmania, using the methods described here, would not only provide a new understanding of the breadth odevil diets but also how far their ecological influence stretches.

As discussed above (see Introduction), cats cause large perturbations to small mammal and bird populations in Australia (Murphy et al., 2019; Woinarski et al., 2018). There is some evidence to suggest that devils suppress cats in Tasmania in a top carnivore mesopredator trophic cascade (Cunningham, Johnson, & Jones, 2019). In essence, the theory suggests that as a top carnivore, devils will exert a competitive and possibly predatory pressure on cats reducing their activity in the presence of devils (Cunningham, Johnson, & Jones, 2019). Interestingly, cat DNA was detected in 14% of Maria Island devil scats. At present, there are no published works with cats appearing as diet items for devils nor direct sightings of devils preying on cats. Cats are present on Maria Island, having been introduced by Europeans, but as population estimates are not made for this species, it is unclear whether devils have had any impact on cat densities. However, camera trap surveys showed a large reduction in cat activity around a prominent short‐tailed shearwater colony from 2013 to 2016 as the devil population on Maria Island grew, suggesting a suppressive effect of devils on cat predation (Scoleri et al., 2020). Examinations of devil diets from across Tasmania more broadly may assist in determining the ecological relationship between feral cats and the native devil.

Broadly, the diet of Maria Island devils was comparable to those from wild Tasmania (Pemberton et al., 2008). The devil has been described as a generalist, both in terms of habitat use from coastal to subalpine regions (Pemberton, 2019) and diet, scavenging and opportunistically preying on a wide variety of species including fish, mammals, birds, and livestock (Pemberton et al., 2008). Habitat and diet generalists are thought to be better adapted to environmental stochasticity than specialists (Clavel et al., 2011). When colonizing a new environment, generalists are considered the most efficient invaders (Wright et al., 2010). For example, the wild boar (*Sus scrofa*) is considered one of the world's best invaders having now colonized every continent excluding Antarctica (Lowe et al., 2000). Generalist habitat and dietary requirements are considered the primary reasons the wild boar is such a successful invader (Senior et al., 2016). Our results, showing an eclectic diet for Maria Island devils, support our suggestion that the generalist and scavenging nature of devils likely contributed to their successful integration into the Maria Island ecosystem.

While we have identified a broad range of species consumed by devils on Maria Island, we cannot definitively say whether these items were consumed via predation or scavenging. This distinction is important to consider when trying to quantify the impact of carnivores on prey species. However, when coupled with population density data, inferences may still be made about whether consumption by carnivores, either predation or scavenging, is contributing to any observed changes in population trends (Egeter et al., 2019). For instance, while our study shows that devils largely consumed macropods, population densities of Forester kangaroos, Bennett's wallabies, and Tasmanian pademelons are either increasing or have remained stable since 2013 (Ingram, 2019). Combined with these estimates, our dietary analysis suggests that devils are not affecting the population densities of macropod species. In contrast, surveys of little penguin and short‐tailed shearwater colonies saw both species below detectable levels at two primary colony sites in 2016 (STDP unpubl. data). Our results confirm that these species were important diet items for devils, postrelease to the island (FOO >25%). As large population declines have been noted for these species, our results are consistent with claims that devils are causing large population declines of little penguins and short‐tailed shearwaters on Maria Island (Wise et al., 2019). It should be noted that neither short‐tailed shearwaters nor little penguins are of global conservation concern (IUCN, 2022). Procellariiform consumption was significantly higher in summer (FOO 54%) than winter (FOO 13%; Table 2). As our sampling was relatively similar between the summer and winter months, the pattern in increased seabird consumption in summer is unlikely to be driven simply by more summer samples and therefore greater detection of seabirds. This observed difference is likely due to the fact that summer is when birds are incubating eggs and hatchlings are emerging which are more vulnerable to predation (Wooller et al., 1990). Indeed, camera trap surveys showed an increase in devil activity around shearwater colonies during their breeding season (Scoleri et al., 2020). In addition, breeding season can be correlated with higher mortality in seabirds (Furness & Birkhead, 1984; Kokko et al., 2004) which could result in increased scavenging of carcasses by devils. Our results are consistent with previous assessments that short‐tailed shearwaters and little penguins could benefit from further protective measures against devils, in addition to the installation of penguin igloos and reduction of devil population size (Wise pers. com.), such as fencing and artificial nest boxes that devils cannot dig into (Scoleri et al., 2020), during the summer months (Peck et al., 2008). Results obtained from testing the effectiveness of such mitigation will be informative in the planning of any future translocations if important populations of ground‐nesting birds are present.

Aligning our dietary observations with previous ecological data suggests that the varied responses to the devil introduction by different species may be associated with the introduction of new predatory pressure from devils. We can compare our study of devil scats collected in 2016–2017 to a previous study of scats collected approximately three years earlier in 2012–2014 (Rogers et al., 2016), and observe that brushtail possum (*Trichosurus vulpecula*) presence in scats has decreased over this period (from 29% to 2%, respectively [Rogers et al., 2016, current study]). It is important to note that morphological and molecular dietary methods are not directly comparable in their ability to reliably detect species across samples (Granquist et al., 2018; Norgaard et al., 2021). The decrease in possum detections in our study could be an artifact of methodological differences between these studies. However, when considering these findings alongside previous ecological observations of a change in brushtail possum behavior to increased risk sensitivity without a change in possum numbers (Cunningham et al., 2019) and a reduction of possum sightings at sea bird colonies as devil numbers increased (Scoleri et al., 2020), we could infer that the possum behavioral change can explain the species being less frequently consumed by devils.

As a preliminary study, our data only covers one full year of devil diets on Maria Island and as such cannot be used to fully quantify seasonal variation in consumption patterns. However, using our available data we showed some interesting patterns that warrant further investigation over several years. For diet items with sufficient data to compare devil consumption patterns between summer and winter, most consumed taxa showed no statistically significant differences across seasons. However, there were some notable patterns. Tetraodontiform consumption was significantly higher in winter samples (FOO 80%) than summer (FOO 32%; Table 2). During May of 2017 when 91% (41/46) of the winter samples were collected, we observed a large die‐off event of one Teraodontiform species, likely resulting in above average consumption of this order. In addition, this die‐off event would have provided an excellent food resource for seabirds, which could have inflated the apparent consumption of Teraodontiform species by devils via meta‐prey consumption. While Diprotodonts were consistently important diet items for devils across summer (FOO 98%) and winter (FOO 98%), Forester kangaroos were consumed significantly more in winter than summer (Table 2). The increased consumption of Forester kangaroos in winter is possibly driven by death of some individuals, as feed availability for kangaroos decreases in winter (Ingram, 2019) and devils feed on the carcasses. Species consumed at lower frequencies were typically more prevalent in summer. In summer, 13 additional species were consumed compared to winter, all with an FOO of ≤10%. These species were mainly fish and reptile species whose activities are generally higher in the warmer months (Spence‐Bailey et al., 2010) likely increasing their interactions with devils. In addition, it is plausible that smaller skink and washed‐up fish species may be accessible diet items for small devils in summer, when devil juveniles are becoming independent (Guiler, 1970). As the ground samples could not be assigned to individuals and, therefore, age class, we did not have sufficient data to compare differences in consumption between juvenile and adult devils. Future dietary analyses across age classes of devils would help to understand how devil diets change as they age.

As *in situ* threat mitigation fails to keep pace with population declines, assisted colonizations and other translocations will become increasingly relevant. Our results suggest that a generalist carnivore, like devils, will adapt well to a novel environment with highly diverse fauna. However, they may cause disruptions to population dynamics of preferred diet species. Taken together, our results indicate that while translocations of carnivores to suitable habitats can be very successful, commonly consumed species should be closely monitored to identify any vulnerable species in need of remedial management, particularly seasonal variations in diet preferences.

## AUTHOR CONTRIBUTIONS

**Elspeth A. McLennan:** Formal analysis (lead); Investigation (equal); Funding acquisition (supporting); Methodology (lead); Writing – original draft (lead); Writing – review & editing (equal). **Phil Wise:** Data curation (equal); Investigation (equal); Writing – review & editing (supporting). **Andrew V. Lee:** Data curation (supporting); Investigation (supporting); Visualization (supporting); Writing – review & editing (supporting). **Catherine Grueber:** Formal analysis (supporting); Methodology (supporting); Supervision (supporting); Writing – review & editing (equal). **Katherine Belov:** Conceptualization (equal); Funding acquisition (equal); Project administration (equal); Supervision (equal); Writing – review & editing (equal). **Carolyn J. Hogg:** Conceptualization (equal); Data curation (supporting); Funding acquisition (equal); Project administration (lead); Supervision (equal); Writing – review & editing (equal).

## CONFLICT OF INTEREST

The authors declare they have no conflict of interest.

## Data Availability

Raw sequence data (fastq.gz) are accessed in Dryad (https://doi.org/10.5061/dryad.2v6wwpzr1).

## APPENDIX 1

**TABLE A1:** Taxonomic information for the species (*N* = 44) consumed by devils on Maria Island including the common name, scientific name, family, order, count, and frequency of occurrence (FOO) of each species

Common name	Scientific name	Family	Order	Count	FOO
Australasian grebe	*Tachybaptus novaehollandiae*	Podicipedidae	Podicipediformes	1	1.0
Australian magpie	*Gymnorhina tibicen*	Artamidae	Passeriformes	3	3.1
Australian sardine	*Sardinops sagax*	Clupeidae	Clupeiformes	5	5.2
Bennett's wallaby	*Macropus rufogriseus*	Macropodidae	Diprotodontia	89	92.7
Black rat	*Rattus rattus*	Muriade	Rodentia	3	3.1
Blotched blue‐tongue lizard	*Tiliqua nigrolutea*	Scincidae	Squamata	21	21.9
Blue Grenadier	*Macruronus novaezelandiae*	Merlucciidae	Gadiformes	1	1.0
Blue mackerel	*Scomber australasicus*	Scombridae	Scombriformes	7	7.3
Brown fur seal	*Arctocephalus pusillus*	Otariidae	Carnivora	2	2.1
Brown thornbill	*Acanthiza pusilla*	Acanthizidae	Passeriformes	2	2.1
Cat	*Felis catus*			13	13.5
Chocolate wattled bat	*Chalinolobus morio*	Felidae	Carnivora	1	1.0
Common brushtail possum	*Trichosurus vulpecula*	Phalangeridae	Diprotodontia	10	10.4
Common ringtail possum	*Pseudocheirus peregrinus*	Pseudocheiridae	Diprotodontia	41	42.7
Common seadragon	*Phyllopteryx taeniolatus*	Syngnathidae	Syngnathiformes	1	1.0
Common wombat	*Vombatus ursinus*	Vombatidae	Diprotodontia	30	31.3
Eastern spinebill	*Acanthorhynchus tenuirostris*	Meliphagidae	Passeriformes	4	4.2
Eastern three‐lined skink	*Acritoscincus duperreyi*	Scincidae	Squanata	2	2.1
Forester kangaroo	*Macropus giganteus*	Macropodidae	Diprotodontia	53	55.2
Green rosella	*Platycercus caledonicus*	Psittaculidae	Psittaciformes	9	9.4
Grey fantail	*Rhipidura fuliginosa*	Rhipiduridae	Passeriformes	1	1.0
Kelp gull	*Larus dominicanus*	Laridae	Charadriiformes	2	2.1
Little egret	*Egretta garzetta*	Ardeidae	Pelecaniformes	1	1.0
Little penguin	*Eudyptula minor*	Spheniscidae	Sphenisciformes	28	29.2
Long‐nosed potoroo	*Potorous tridactylus*	Potoroidae	Diprotodontia	58	60.4
Ornate cowfish	*Aracana ornata*	Aracanidae	Tetraodontiformes	1	1.0
Rough leatherjacket	*Scobinichthys granulatus*	Monacanthidae	Tetraodontiformes	1	1.0
Scarlet robin	*Petroica boodang*	Petroicidae	Passeriformes	3	3.1
Senator wrasse	*Pictilabrus laticlavius*	Labridae	Labriformes	1	1.0
Short‐beaked echidna	*Tachyglossus aculeatus*	Tachyglossidae	Monotremata	5	5.2
Short‐finned pilot whale	*Globicephala macrorhynchus*	Delphinidae	Artiodactyla	1	1.0
Short‐tailed shearwater	*Puffinus tenuirostris*	Procellariidae	Procellariiformes	33	34.4
Silvereye	*Zosterops lateralis*	Zosteropidae	Passeriformes	2	2.1
Southern boobook	*Ninox novaeseelandiae*	Strigidae	Strigiformes	11	11.5
Southern brown bandicoot	*Isoodon obesulus*	Peramelidae	Peramelemorphia	10	10.4
Starling	*Sturnus vulgaris*	Sturnidae	Passeriformes	2	2.1
Swamp rat	*Rattus lutreolus*	Muridae	Rodentia	1	1.0
Tasmanian blenny	*Parablennius tasmanianus*	Blenniidae	Blenniiformes	1	1.0
Tasmanian pademelon	*Thylogale billardierii*	Macropodidae	Diprotodontia	45	46.9
Tasmanian scrubwren	*Sericornis humilis*	Acanthizidae	Passeriformes	1	1.0
Tasmanian tree skink	*Niveoscincus pretiosus*	Scincidae	Squamata	1	1.0
White's skink	*Liopholis whitii*	Scincidae	Squamata	1	1.0
Yellow wattlebird	*Anthochaera paradoxa*	Meliphagidae	Passeriformes	1	1.0
Yellowfin leatherjacket	*Meuschenia trachylepis*	Monacanthidae	Tetraodontiformes	2	2.1
